# Echocardiographic evaluation of a single bolus of erythropoietin effects on reducing ischemia-reperfusion injuries during coronary artery bypass graft surgery. A randomized, double blinded placebo control study

**DOI:** 10.1186/1749-8090-8-S1-O208

**Published:** 2013-09-11

**Authors:** SH Ziabakhsh Tabary, R Jalalian, FE Mokhtari, M Habibi

**Affiliations:** 1 Cardiac Surgery Department, Mazandaran Heart Center, Sari, Iran

## Background

erythropoietin (EPO) is known as a regulating hormone for production of red blood cells called Erythropoiesis. Some studies have shown that erythropoietin have some non-hematopoietic protective effects on ischemia-reperfusion injury in myocardial cells. We evaluated the effect of exogenous EPO infusion on reducing ischemia-reperfusion injuries and improvement of cardiac function by echocardiography shortly after coronary artery bypass graft surgery.

## Methods

43 patients were joined the study and randomly divided in two groups, EPO group: receiving standard medication and CABG surgery plus 700 IU/kg erythropoietin (PD Poietin, puyeshdaroo, Iran) and control group: receiving standard medication and CABG surgery plus 10cc normal saline as placebo. The cardiac function was assessed by Echocardiography in all patients in before, 4 days after and also 30 days after CABG operation.

## Results

Echocardiography indicated that EF had no differences between EPO and control group at 4 days (47.05±6.29 vs 45.90±4.97, P=0.334) or 30 days after surgery (47.27±28 vs 46.62±5.7, P=0.69). There were no differences between EPO and control group in wall motion score index at 4 days (P=0.83) or 30 days after surgery (P=0.902). In EPO group: Left ventricle end systolic and diastolic diameter (LVESD, LVEDD) had reduction, as compared to control group.

## Conclusions

Our data suggest that peri-operatively exogenous EPO infusion can’t improve ventricular function and Wall motion index in first weeks after surgery. But as compared to control group, reduction in LVEDD and LVESD at 4 days or 30 days after CABG surgery in EPO group suggested that EPO had correlation with reduction of myocytes remodeling and reperfusion injury early after CABG surgery.

